# Radiological diagnosis of perinephric pathology: pictorial essay 2015

**DOI:** 10.1007/s13244-016-0536-z

**Published:** 2017-01-03

**Authors:** Goran Mitreski, Tom Sutherland

**Affiliations:** 1 University Hospital Geelong, Bellarine Street, 3220 Geelong, Australia; 2 Medical Imaging Department, St Vincent’s Hospital, 41 Victoria Pde, 3065 Fitzroy, Australia

**Keywords:** Kidney, Neoplasms, connective and soft tissue, Tomography, spiral computed, Magnetic resonance imaging, Perinephric space

## Abstract

**Abstract:**

The perinephric space, shaped as an inverted cone, sits between the anterior and posterior renal fasciae. It can play host to a variety of clinical conditions encountered daily in the reporting schedule for a radiologist. Lesions may be classified and diagnosed based on their imaging characteristics, location and distribution. A broad range of differential diagnoses can be attributed to pathology sitting within this space, often without clinical signs or symptoms. An understanding of commonly encountered conditions affecting the perinephric space, along with characteristic imaging findings, can illustrate and often narrow the likely diagnosis. The aim of this essay is to describe commonly encountered neoplastic and non-neoplastic entities involving the perinephric space and to describe their key imaging characteristics.

**Teaching Point:**

• *Despite often a bulky disease, perinephric lymphoma does not produce obstruction or stenosis*.

• *In primarily fatty masses, defects within the renal capsule likely represent angiomyolipoma*.

• *Consider paraganglioma if biopsy is planned; biopsy may lead to catecholamine crisis*.

## Introduction

A diverse assortment of pathologies can present within the perinephric space. The aetiology of disorders seen on radiological imaging can be attributed to solid tumours, both benign and malignant; fluid collections; inflammatory masses; or proliferative syndromes. Appreciation of the close relationship between the perinephric space and adjacent retroperitoneal organs and planes can assist in localising the site of origin of pathologies and can help narrow the differential diagnosis. An understanding of the imaging characteristics of these pathologies will then enable a more accurate diagnosis and guidance of treatment.

This pictorial essay will review and illustrate conditions commonly encountered within the perinephric space, outlining relevant anatomical landmarks, radiological signs and features, along with patterns of disease spread.

### Anatomical landmarks

**Fig. 1:**
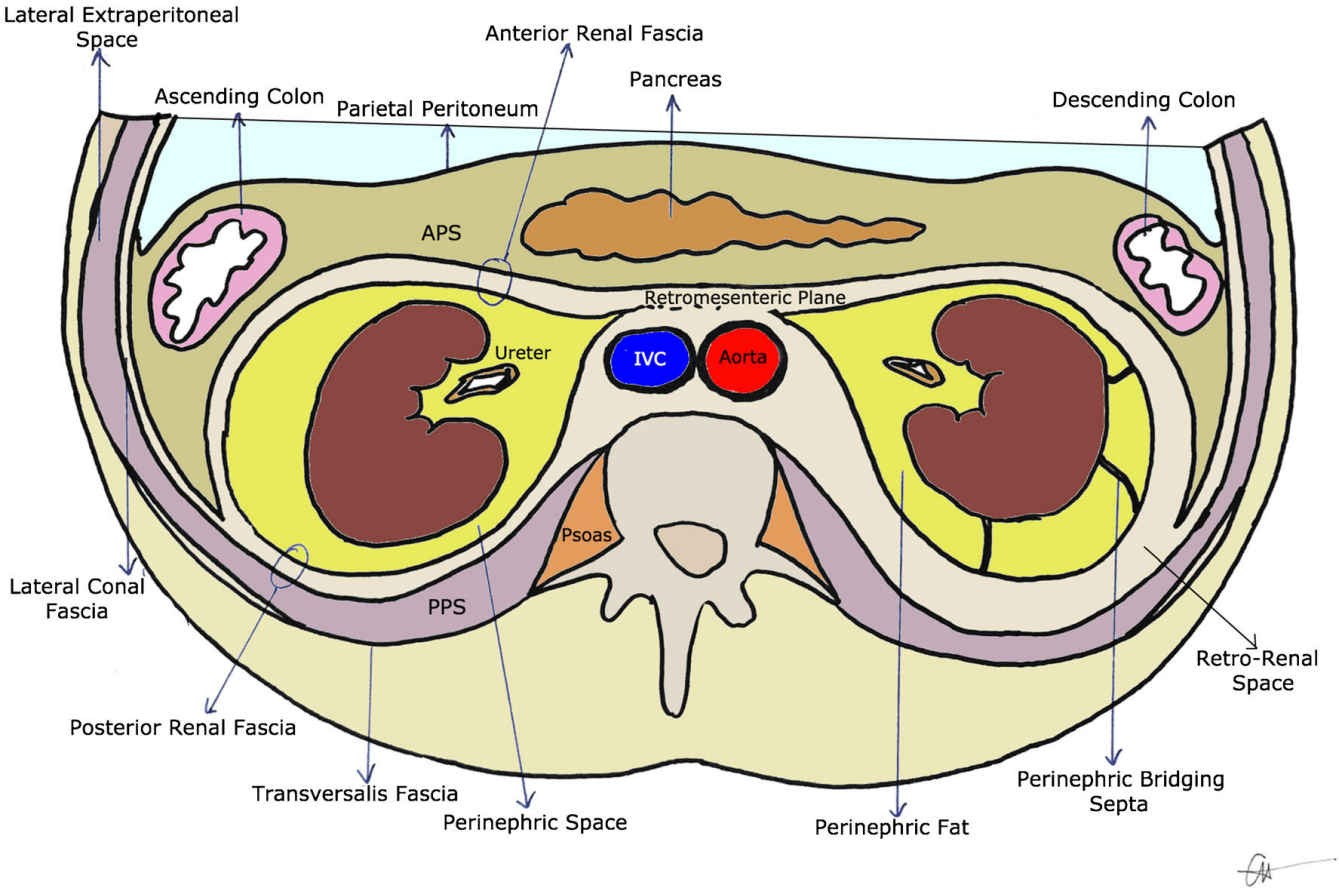
Diagrammatic representation of the perinephric space. The posterior pararenal space (PPS) and anterior pararenal space (APS) have been exaggerated to provide representation of their relation to other retroperitoneal structures. Perinephric bridging septa are seen between the left kidney and the adjacent renal fascia

Basic knowledge and understanding of the retroperitoneal anatomical spaces is essential to understanding disease presentation and patterns of spread. The retroperitoneum is divided into three distinct compartments: the perinephric space, anterior pararenal space and posterior pararenal space [1] (Fig. 1).

The perinephric space is formed by adjacent bordering pararenal spaces anteriorly and posteriorly. The perinephric space contains the kidneys, proximal ureters, adrenal glands, fat, bridging septa (Kunin’s septa), vessels and lymphatics [2]. The thin lamina anteriorly, also known as Gerota’s fascia, fuses with the thicker posterior Zuckerkandl’s fascia to form the lateral lateroconal fascia. Medially, the perinephric fascial extension joins the periureteral connective tissue and duodenal attachments on the right, whilst on the left its attachment is seen to extend towards the periaortocaval connective tissue. Superiorly, it attaches to the diaphragm, whilst it continues and attaches to the iliac fascia inferiorly as a combined inter-fascial plane [3].

**Fig. 2:**
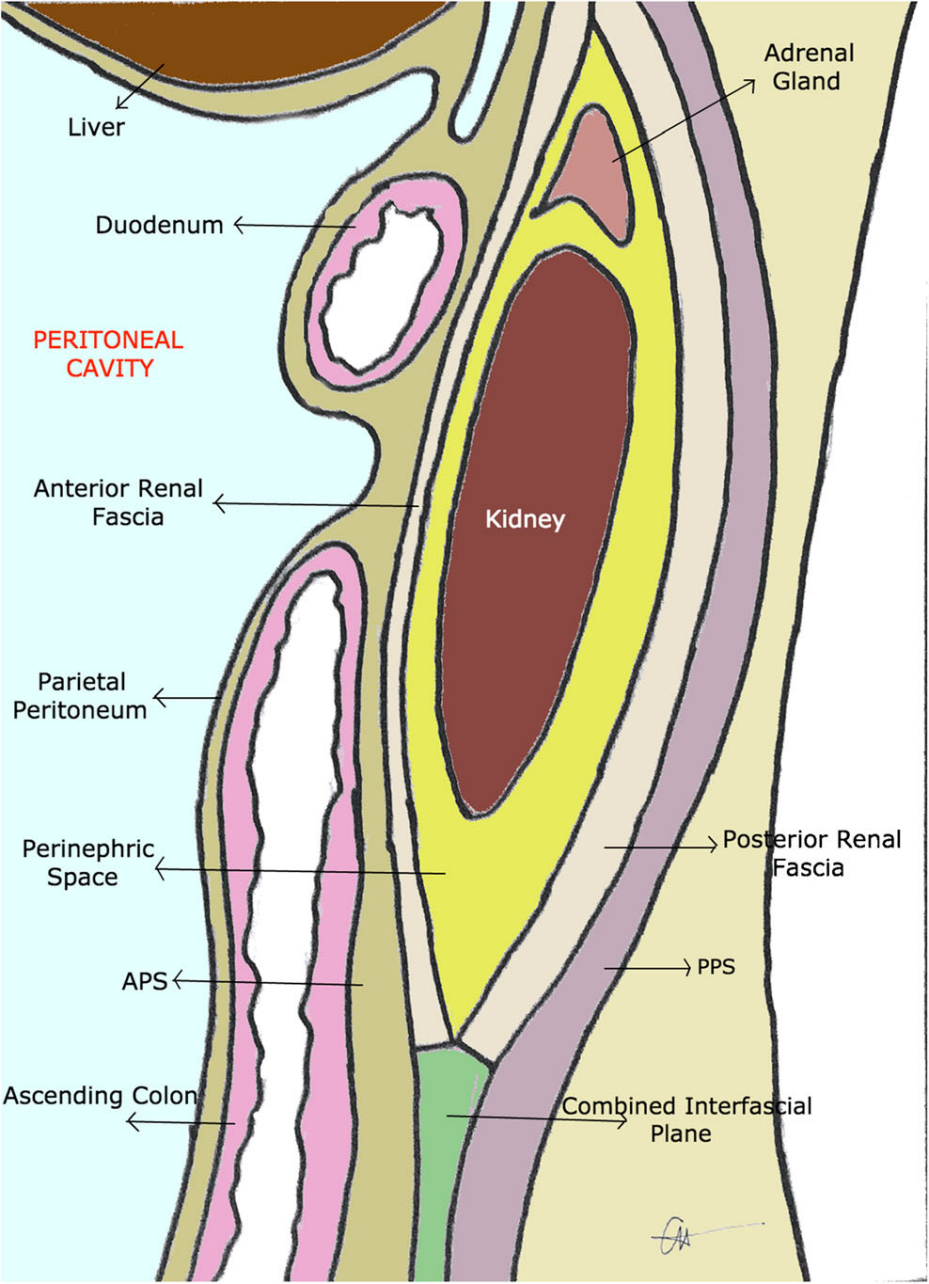
Lateral longitudinal representation of the perinephric space. The adrenal gland lies within the perinephric space, superior to the kidney. The anterior renal fascia and posterior renal fascia join inferiorly into the pelvis as the combined inter-fascial plane

**Fig. 3:**
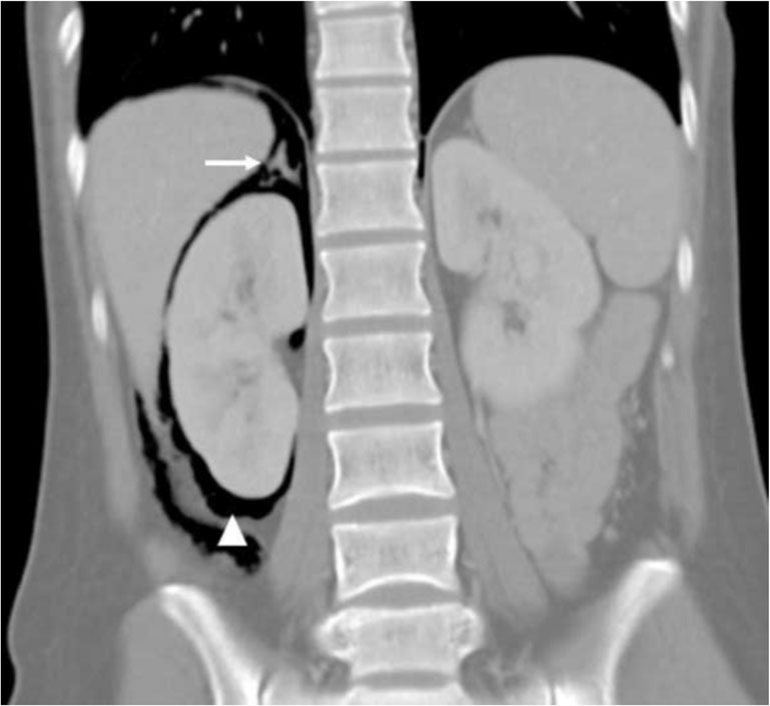
Conal portal venous CT shows gas in the perinephric space (*arrowhead*), demonstrating its longitudinal conal orientation and that it contains both the kidney and the adrenal (*arrow*). The gas was secondary to a perforation during colonoscopy

The perinephric space appears as an inverted cone, corresponding to the embryological migration of the renal system from the pelvis [4] (Figs. 2 and 3).

The pararenal spaces lie superficial to the perinephric space. The anterior pararenal space lies between the parietal peritoneum and Gerota’s fascia, and contains the pancreas, duodenum, and ascending and descending colon. The posterior pararenal space sits between Zuckerkandl’s fascia and the transversalis fascia, and contains no organs, only fat pads. As the inter-fascial plane continues towards the iliac fascia, the space produced between Gerota’s fascia and Zuckerkandl’s fascia is known as the infrarenal space [1, 3].

As the perinephric space lies centrally in the retroperitoneal space, disease manifestation and transmission of pathology, such as that of malignancy, can traverse fascial planes, as can rapidly accumulating fluid collections [2]. Slowly accumulating fluids are usually confined to their compartment of origin.

### Non-neoplastic

#### Blood

A perinephric haematoma is typically described as fluid that is confined within the dense, collagenous Gerota’s fascia [5]. Traumatic causes include both blunt and penetrating injury, while iatrogenic causes include renal biopsy, ablation, nephrostomy and lithotripsy complications. These causes are typically distinguishable by the clinical setting or the presence of coexisting injuries such as splenic or hepatic lacerations [6]. Spontaneous bleeding can occur in hypo-coagulable states, but is more frequently a complication of primary renal neoplasms such as angiomyolipoma or renal cell carcinoma. A bleeding tumour can usually be easily identified with cross-sectional imaging, although a small lesion may occasionally be obscured by the haematoma in the acute setting. In these cases, follow-up will be required to re-examine the kidney once the haematoma involutes. Angiomyolipomas can be differentiated from renal cell carcinoma based upon the presence of macroscopic fat within them, and their haemorrhagic risk is greatest when they are over 4 cm in size [7]. Spontaneous bleeding can also occur secondary to polyarteritis nodosa, vascular malformations or aneurysms arising from the renal arteries or from the abdominal aorta [8].

**Fig. 4:**
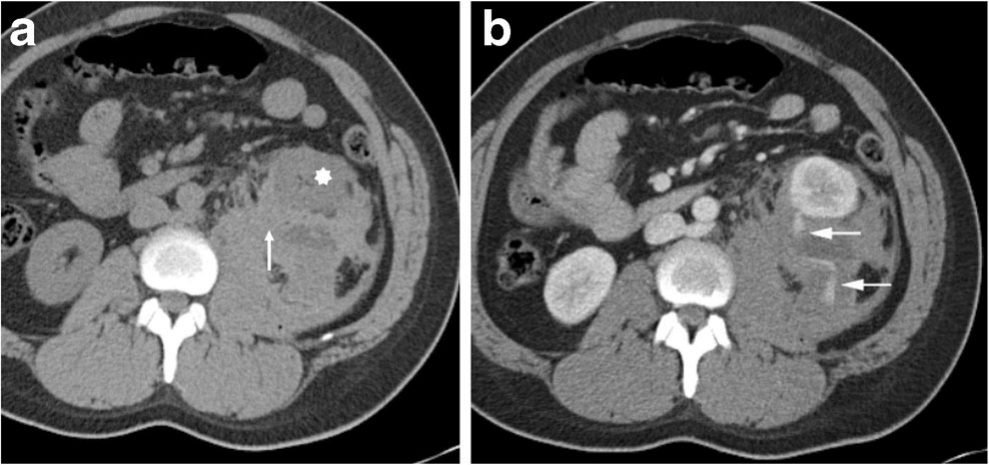
**a** Acute left-sided haematoma that is isodense to muscle (*arrow*), displacing the kidney anteriorly (*star*). **b** The portal venous phase shows active bleeding (*arrows*) into the haematoma. The bleed was secondary to an ultrasound-guided biopsy

Acute haematoma has a higher attenuation value than renal parenchyma on unenhanced CT imaging (Fig. 4a) and a lower attenuation value on contrast-enhanced imaging (Fig. 4b). Active bleeding can be detected using multiphase CT. Subacute haematomas have a Hounsfield density closer to that of water (0 to +20 HU) secondary to haematoma liquefaction, and occasionally a fluid level can be appreciated with the denser element being dependent [8, 9]. The aetiology of haematoma formation is outlined in Table 1 [10, 11].

**Table 1:** Aetiology of perinephric haematoma

Traumatic	Blunt trauma
	Penetrating injury
Iatrogenic	Renal biopsy
	Surgery: nephron-sparing/partial nephrectomy
	Nephrostomy insertion
	Ablation
	Lithotripsy
Spontaneous	Bleeding diathesis (hepatic failure, haemophilia, systemic lupus erythematosus, thrombocytopaenia) medication-related
	Vascular disease (ANCA vasculitis, Behçet’s disease, arterial venous malformation)
	Renal tumours (angiomyolipoma, renal cell cancer, renal metastases)
	Renal infarction (emphysematous pyelonephritis, abscess)
	Adrenal haemorrhage (sepsis, burns, complicated pregnancy)
	Renal cysts

#### Urine

**Fig. 5:**
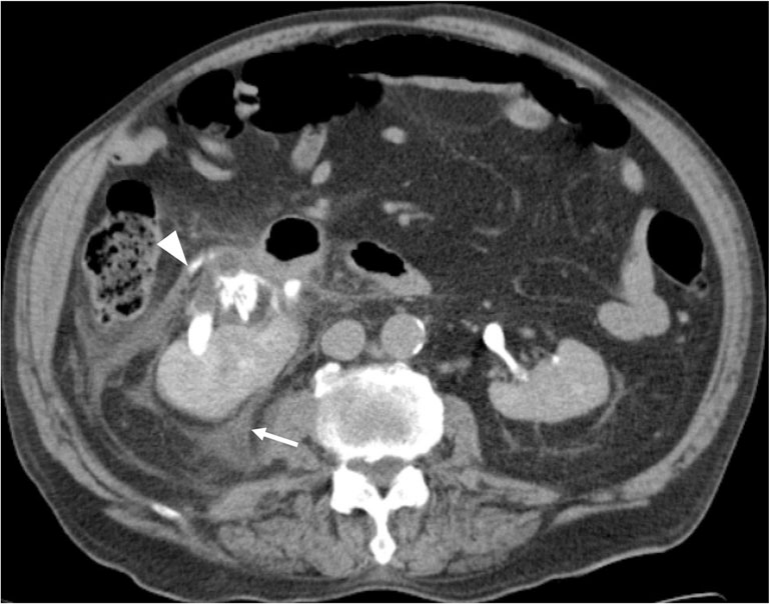
Delayed-phase CT in a patient with calyceal rupture following lithotripsy shows fluid in the perinephric space (*arrow*) and extravasation of contrast (*arrowhead*) in keeping with active urine leak

**Fig. 6:**
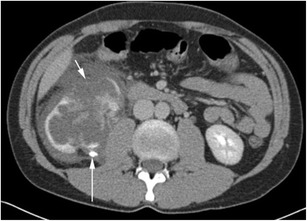
Delayed-phase CT shows fluid filling the right perinephric space in a patient following blunt trauma. The kidney has been lacerated (*short arrow*), and urinary contrast extravasation is shown posteriorly (*long arrow*)

**Fig. 7:**
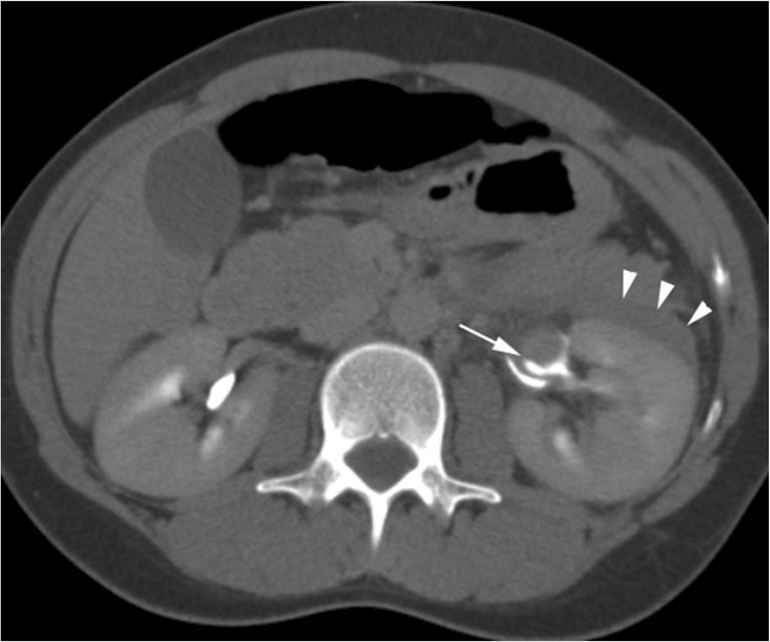
A delayed-phase CT shows a left renal pelvis with extravasation of urine anterior to it (*arrow*). Note the unopacified urine in the perinephric space (*arrowheads*)

Urine leaks are most frequently secondary to trauma (Fig. 5), either blunt or penetrating, and most often arise proximally from the calyces or renal pelvis, and therefore present as fluid within the perinephric space [12]. These points of relative weakness are also the sites of rupture when obstructive back pressure is the cause of system rupture. The primary site of obstruction may be more distally located in the urinary system and may relate to calculi, retroperitoneal fibrosis, pregnancy or other pelvic masses. Iatrogenic causes are recognised but are relatively uncommon except for ureteral injuries, with the site of leak relating to the site of ureteral intervention/injury [13]. Urine on contrast-enhanced CT has a Hounsfield unit (HU) nearing that of water (0 to +10), identical to other simple fluid collections. Delayed-phase contrast CT can show extravasation of urine (Fig. 6), allowing accurate differentiation from other fluid collections, and multiplanar reformations can aid in identifying the precise site of urine leak (Fig. 7). Alternatively, in patients with renal impairment precluding the use of intravenous contrast, renal scintigraphy can be used to demonstrate communication between the renal collecting system and the perinephric fluid, albeit with reduced spatial resolution. It is important to remember to inspect the entire urinary system to identify the primary cause of leakage, especially in cases secondary to ureteral obstruction. In cases of diagnostic dilemma, fine needle aspiration with measurement of fluid creatinine can be diagnostic [3].

The transition of acute urinary extravasation to chronic urinoma formation is secondary to encapsulated urine that leaks and collects within the boundaries of the anterior and posterior renal fascia, producing a proinflammatory and fibrosing state. A urinoma may present with gross fibrous thickening and an enhancing rim and septa on contrast-enhanced CT [3].

#### Lymph

**Fig. 8:**
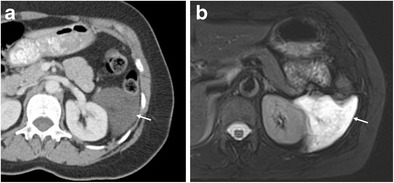
**a** A well-defined and fluid attenuation homogeneous cystic lymphangioma in the left perirenal space (*arrow*), with no capsule and no evidence of mass effect on the adjacent kidney. **b** A T2-weighted MRI shows the fluid signal of the lesion (*arrow*)

Lymphangiomas are congenital benign cystic tumours that most frequently occur within the neck, although up to 5% occur within the abdominal cavity, with the mesentery and retroperitoneum the most common abdominal sites [14, 15]. When small, they are asymptomatic, often diagnosed as an incidental finding during imaging examinations for other indications (Fig. 8a). When large, they can present with mass effect due to compression of adjacent organs, and similar to their cervical counterparts can be trans-spatial. Lymphangiomas comprise a series of dilated cystic spaces that contain fluids such as chyle, blood products from previous haemorrhage, serous fluid or a mixture. The fluid typically outlines the thin enhancing walls of the cystic spaces, and at MRI, the lesions follow fluid signal intensity on all acquisitions unless complicated by blood product or superimposed infection [16] (Fig. 8b). Elevated apparent diffusion coefficient (ADC) values are also typical.

**Fig. 9:**
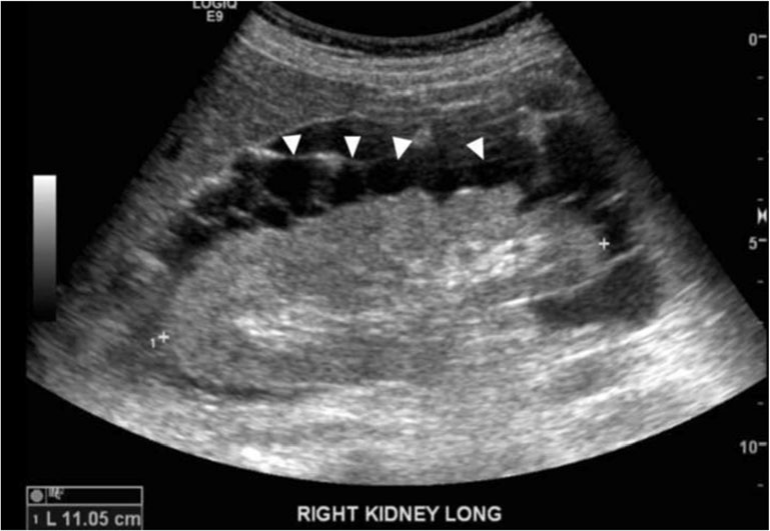
Ultrasound image showing multiple subcapsular cystic lesions (*arrowheads*) bulging into the perinephric space outlining the entire kidney in a patient with lymphangiectasia

Sonographically, the lesions appear as anechoic fluid divided by thin-walled septa (Fig. 9), with no appreciable flow on Doppler imaging. Differentiation from other fluid collections can be difficult, as can diagnosing superimposed infection. Aspiration typically yields a milky white fluid, with elevated triglyceride level [17].

Renal lymphangiomatosis is a rare condition of unknown aetiology. It is believed to arise secondary to poor renal lymphatic communication during embryological development. It is characterised by small peri-pelvic and capsular cystic changes that may be uni- or multilocular. The imaging appearance is similar to lymphangiomas, apart from the location as a subcapsular pathology rather than arising within the perinephric space. The condition may be unilateral or bilateral. It can occur at any age and has no predilection for sex. Diagnosis tends to focus on histology, as radiological signs are often indeterminate for lymphangioma [18]. Treatment is usually conservative yet percutaneous drainage or surgical marsupialiation is warranted if fluid shifts and fluid expansion cause mass effect [19, 20].

#### Pseudocyst

**Fig. 10:**
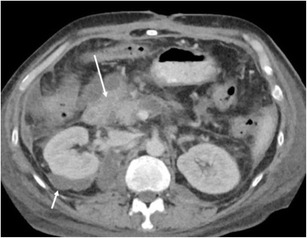
A right perinephric pseudocyst (*short arrow*). Note the substantial inflammation in the anterior pararenal space centred on the pancreas (*long arrow*) in a patient with acute pancreatitis

Acute pancreatitis is accompanied by fluid collections in around 43% of cases [21], with risk factors including alcohol-induced pancreatitis, younger age and severe pancreatitis [6, 22]. Acute peripancreatic fluid collections (APPFCs) occur in the early phase of pancreatitis, may be multiple, are typically sterile and usually spontaneously resolve [23]. When present for over 4 weeks, they become known as pseudocysts. Pseudocyst formation occurs in 5–15% of patients following acute pancreatitis [21, 24]. The collections are homogenous and of fluid density/intensity, and with time can develop smoothly enhancing margins. Imaging will show a rounded or oval fluid-filled collection without any internal septa or non-liquefied components (Fig. 10). The presence of debris and heterogeneity indicates complexity and that the area is an acute necrotic collection/walled-off necrosis, with these lesions usually confined to the anterior pararenal space. Aspiration of APPFCs or pseudocysts reveals elevated levels of amylase [25].

In 40-69% of pseudocysts, a communication will remain with the pancreatic duct [26]. MR cholangiopancreatography is more accurate than contrast-enhanced CT at depicting this residual ductal communication﻿; endoscopic retrograde cholangiopancreaticography (ERCP) remains the gold standard for diagnosis [27]. MRI can also demonstrate blood product within a collection when haemorrhagic transformation is a concern [28].

#### Perinephric abscess

**Fig. 11:**
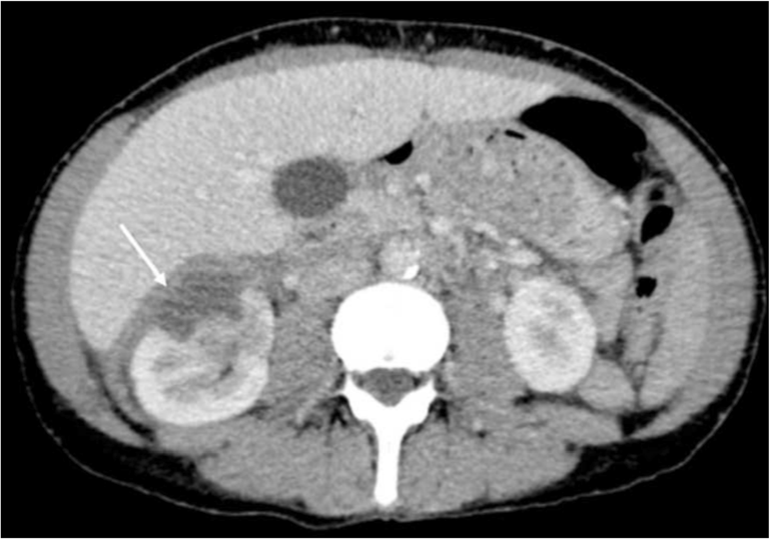
Portal venous CT shows a right renal abscess that has burst into the perinephric space (*arrow*), best appreciated as the soft tissue density interposed between the kidney and adjacent liver

Abscess within the perinephric space can present a diagnostic challenge. Depending on chronicity, aetiology and complications, it may mimic a tumour radiologically if chronic or may mandate evacuation and immediate treatment if acute. Predisposing factors for perinephric abscess include diabetes, pyelonephritis, urinary tract calculi and immunosuppression. Acute perinephric abscess is accompanied by fever, flank pain and leukocytosis. CT and MRI findings are non-specific and include thickening of the adjacent fascia, obliteration of perinephric fat planes and fat stranding [29, 30]. Contrast-enhanced CT imaging may demonstrate rim enhancement with central hypo-attenuation and thickened septa. The presence of thickened perinephric fascia and increased adjacent fat signalling are further suggestive of an infectious process rather than a malignant cystic neoplasm [3, 31] (Fig. 11).

In cases of emphysematous infection, CT can visualise gas extending from the kidney into the perinephric space. Xanthogranulomatous pyelonephritis, a rare progressively destructive process with lipid-rich macrophages replacing degenerative renal tissue, can also present with abscesses within the perinephric space as the inflammatory process spreads [3]. Imaging findings include unilateral enlargement of the kidney, heterogeneous parenchymal enhancement on contrast-enhanced CT imaging, hydronephrosis and obstructive calculi [32].

#### Thickened bridging septa

**Fig. 12:**
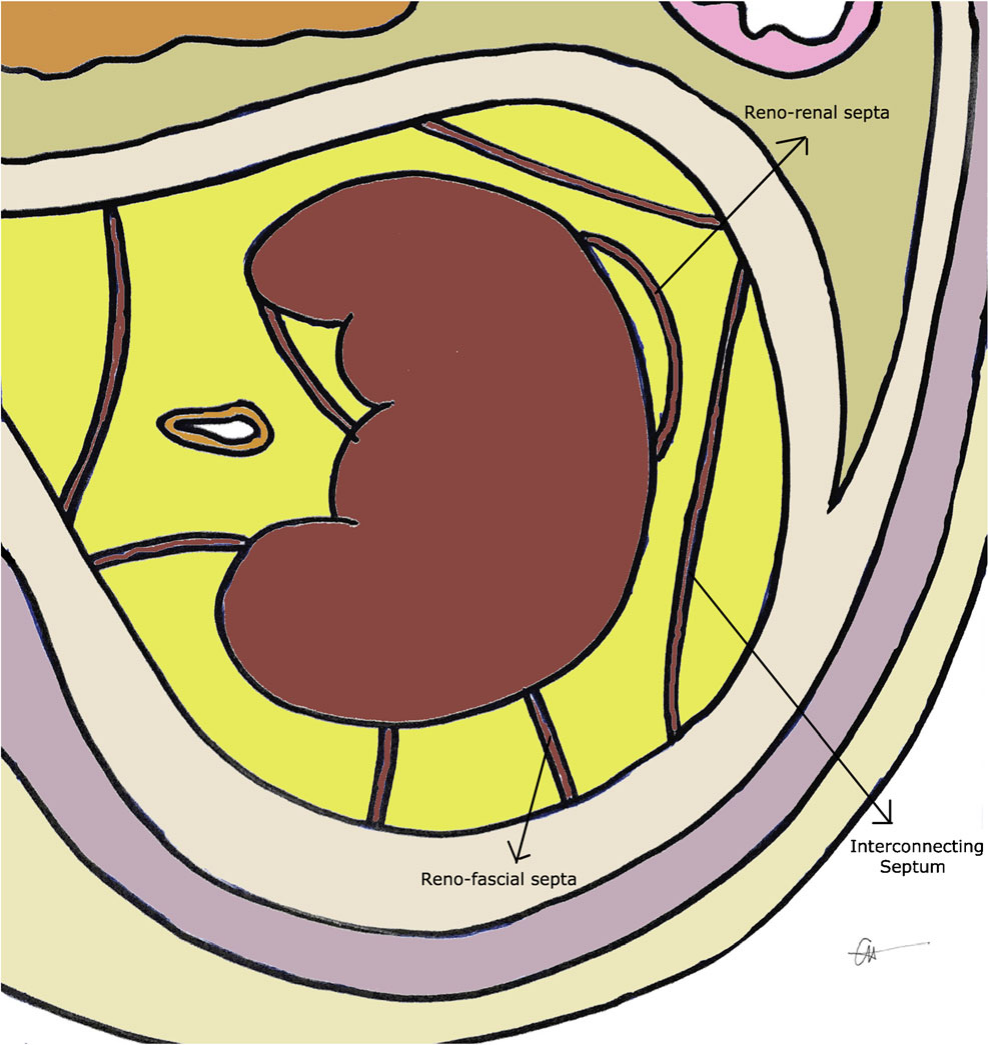
Three types of bridging septa have been described: reno-renal, fibres running parallel to the renal capsule and attaching back onto the kidney; reno-fascial, septa connecting the capsule to the adjacent anterior or posterior renal fasciae; and interconnecting fascia, connecting the anterior and posterior layers of the perinephric fascia

Perinephric bridging septa (PBS) are composed of numerous fibrous lamellae which traverse the perinephric fat, extending from the renal capsule to the renal fascia and to other points on the posterolateral aspect of the capsule (Fig. 1). They serve to suspend the kidneys within the perirenal space [33]. Kunin [34] described three types of septa, as depicted in Fig. 12. PBS may serve as a conduit for the spread of inflammation, fluid or neoplasm from the kidney to the retromesenteric or retrorenal fascial planes, and can assist the radiologist in localising relevant pathology on abdominal imaging. Inflammation originating from the kidney may thicken the PBS and perinephric fascia. Urinary extravasation may thicken the PBS and appear as irregular perinephric or periureteral fluid collections. Fluid tracking along the PBS producing a liner or curvilinear pattern within the perinephric space is often seen in traumatic perinephric haematoma.

#### Retroperitoneal fibrosis

Retroperitoneal fibrosis is characterised by the proliferation of fibro-inflammatory tissue, usually surrounding the infrarenal portion of the great vessels (aorta, inferior vena cava and iliac vessels) [35]. Perinephric fibrosis involving the ureters and kidney can result from extension of the infiltrative soft tissue process, causing hydronephrosis and hydroureter [36]. Imaging features of perinephric fibrosis exhibit a soft tissue infiltrate centred around the great vessels, with possible extension into the perinephric space and rarely into the retrorenal space and retromesenteric spaces [37]. CT imaging can depict fibro-inflammatory tissue with an attenuation coefficient similar to that of psoas muscle on non-contrast imaging, whilst contrast-enhanced CT imaging shows enhancement dependent on the stage of active disease. MRI shows low T1-weighted signalling, with variable T2-weighted signalling, reflecting the degree of active inflammation (hypercellularity and oedema) [36]. The diagnosis of retroperitoneal fibrosis warrants biopsy and tissue sampling, as the disease although mostly idiopathic, can be secondary to a desmoplastic response to retroperitoneal metastases [38, 39].

### Neoplastic

#### Lymphoma

**Fig. 13:**
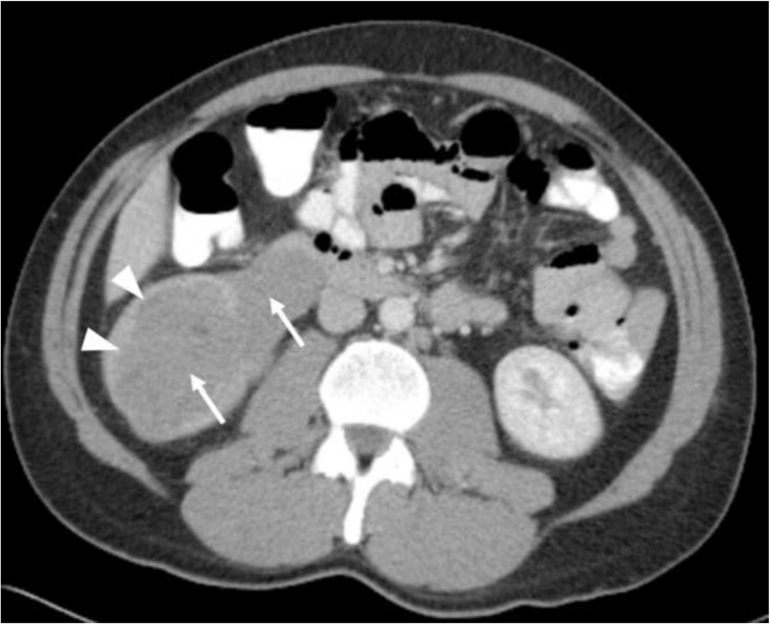
Hypo-enhancing soft tissue fills the right renal pelvis, obliterating sinus fat and propagating along the proximal ureter (*arrows*). The overall reniform shape of the kidney is preserved, and the parenchyma is stretched but not invaded (*arrowheads*)

Renal lymphoma is rarely seen as a primary renal lesion, with incidence of less than 1% [40]. Renal involvement tends to be secondary to systemic non-Hodgkin’s lymphoma, especially B-cell lymphomas. Pathologic studies show that renal involvement is common. However, this is typically on a microscopic level, beyond the resolution of current imaging technology—hence a discord between autopsy rates of 30–60% and imaging manifestations of 1–8% [41–43]. Renal involvement can take the form of diffuse renal infiltration, solitary or multifocal masses, or soft tissue masses within the perinephric space. The perinephric involvement is due to contiguous spread from adjacent retroperitoneal lymph nodes or by extension along the ureter or from the adrenal [44]. The masses are homogenous, and in the absence of treatment, calcification and necrosis are rare. A key imaging feature suggesting the diagnosis is that despite often bulky disease that encases vessels and ureter, lymphoma usually does not produce obstruction (Fig. 13) or significant stenosis [45, 46]. Evidence of involvement at other sites such as the spleen or distant nodal stations should also alert the radiologist to the diagnosis.

At MRI, the masses have an intermediate signal on both T1- and T2-weighted images, although a high T2 signal may occur occasionally [41]. The enhancement may be more heterogeneous than that encountered on CT. The tumours have restricted diffusion, in keeping with small round blue-cell tumours encountered elsewhere.

**Fig. 14:**
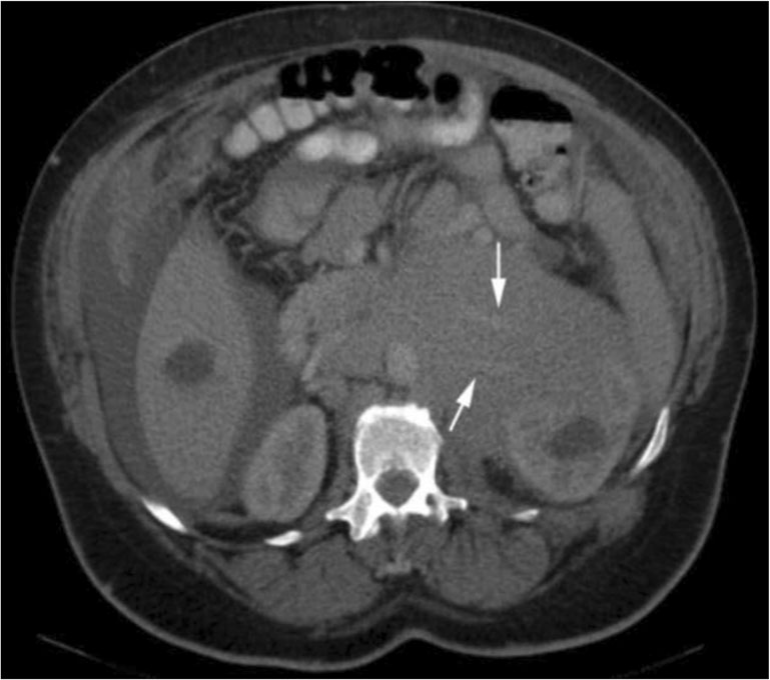
Diffuse large B-cell lymphoma filling the left perirenal space and laterally displacing the kidney. The best clue to the diagnosis is that patent vessels can be seen passing through the mass (*arrows*)

It is usually seen as a spectrum of multi-system lymphoproliferative conditions. The genitourinary system is the second most commonly affected anatomic entity for extra-nodal spread of lymphoma, next to the haematopoietic and reticuloendothelial organs [45, 47]. CT remains the imaging modality of choice, but US, MRI and PET CT all have important diagnostic implications; combination PET/CT has shown staging sensitivity and specificity of up to 97% [48, 49]. The presentation of renal lymphoma can include single or multiple lesions, direct extension from retroperitoneal adenopathy, and diffuse infiltration of one or both kidneys [45]. Renal lymphoma enhances poorly on contrast-enhanced CT (Fig. 14).

#### Liposarcoma

Retroperitoneal sarcomas are rare tumours, and a number of subtypes may occur, the most common of which are liposarcoma and Leiomyosarcoma [50]. Due to the large potential space within the retroperitoneum, and late non-specific symptoms of retroperitoneal sarcomas, they are frequently large at the time of diagnosis. As such, retroperitoneal space involvement carries a poor prognosis, as does tumour grade [51, 52]. This has led to more aggressive surgical resection and radiation therapy, thus requiring an accurate description of the anatomical boundaries of the tumour to guide therapy.

**Fig. 15:**
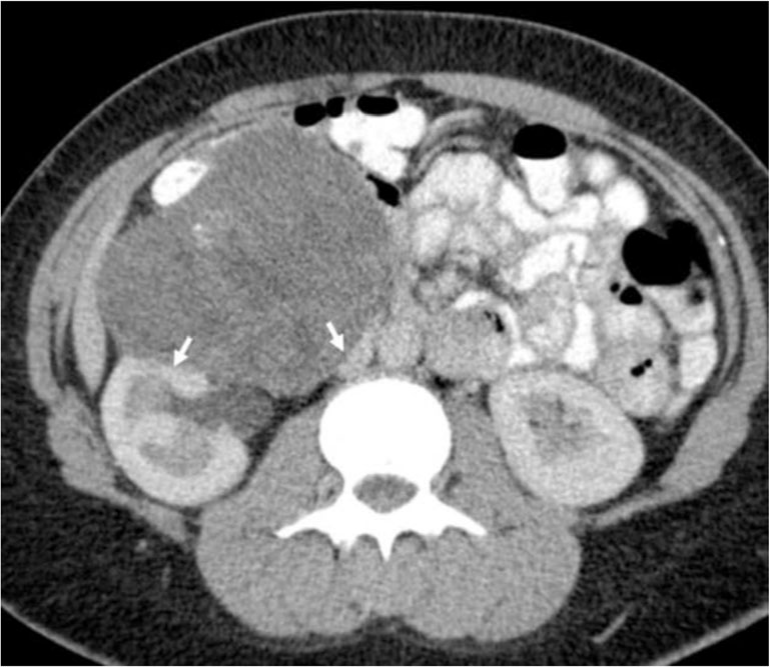
A soft tissue mass indents the kidney and cava (*short arrows*) and displaces gut. Biopsy showed it was a liposarcoma, although no macroscopic fat was evident on imaging

**Fig. 16:**
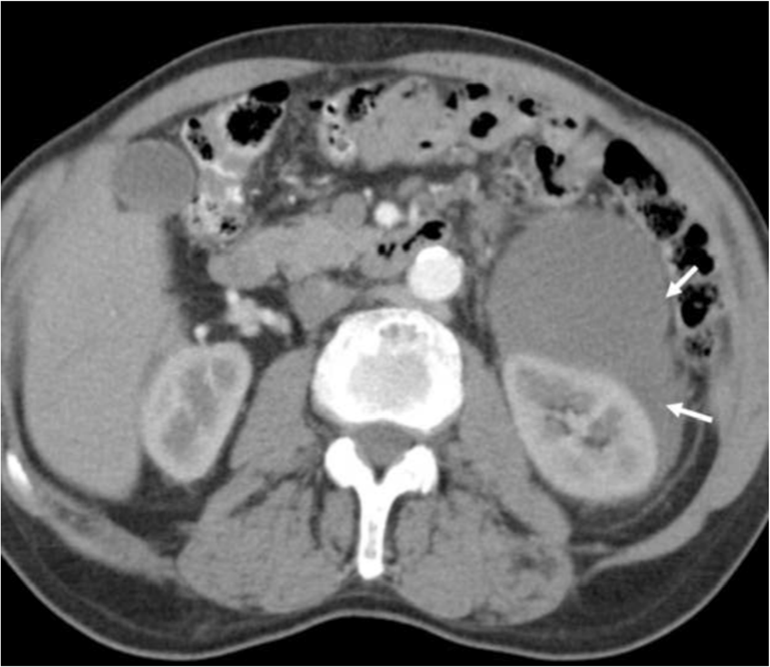
A homogenous low-density myxosarcoma (*arrows*) in the left perirenal space encases the anterolateral kidney

Liposarcoma remains the most common primary retroperitoneal malignancy and the second most common presentation of soft tissue sarcoma after pleomorphic undifferentiated sarcoma [53, 54]. Four subtypes are described: well-differentiated ​or atypical lipomatous tumour  (most common subtype), myxoid including round cell, de-differentiated (least common subtype) and pleomorphic [55]. Imaging findings commonly reflect underlying histological subtype [56, 57]. Lesions that are primarily composed of macroscopic fat tend to be well-differentiated, demonstrating fat attenuation on CT and following fat signal on MRI, with a paucity of soft tissue elements. It is important when performing biopsy to target the soft tissue component of the tumour, as these are frequently the poorly differentiated component [53]. Macroscopically, these tumours show greater than 75% fat composition [58]. De-differentiated tumours will share radiological characteristics with well-differentiated liposarcoma and will have additional focal, nodular non-lipomatous regions greater than 1 cm in size [59]. Calcification or ossification within a liposarcoma has been shown to be a poor prognostic feature [50]. Pleomorphic liposarcomas tend to appear more as soft tissue masses without defining lipomatous characteristics (Fig. 15) and with extensive anaplasia [53]. Myxoid liposarcomas (Fig. 16) can appear cystic and can have elevated T2 signal intensity in non-lipomatous elements, with a tendency for reticular enhancement [60]. Ultrasound can be useful in myxoid subtypes, as sonography can demonstrate that the mass is not truly cystic, and further investigation with biopsy may be warranted.

In well-differentiated primarily fatty masses, it is important to search for a defect within the renal capsule, as when a lesion is present, it most likely represents an angiomyolipoma rather than a sarcoma [61]. The adrenal gland should also be inspected to differentiate the mass from an adrenal myelolipoma.

#### Leiomyosarcoma

Leiomyosarcoma is the second most common primary retroperitoneal malignancy in adults [54]. These tumours can be intravascular (62%), extravascular (5%) or a combination of both (33%) [46]. The tumour origin comprises cells showing distinct smooth muscle features, and retroperitoneal malignancy is believed to originate from vessels, smooth muscle of the renal capsule, renal pelvis, calyxes and embryological remnants within the retroperitoneum [62]. Diagnosis is often late, given the nonspecific clinical findings and the relative accommodation of tumour growth provided by the retroperitoneum. CT and MR imaging tend to demonstrate heterogeneous large tumours with extensive necrotic and cystic change [46].

#### Angiomyolipoma

**Fig. 17:**
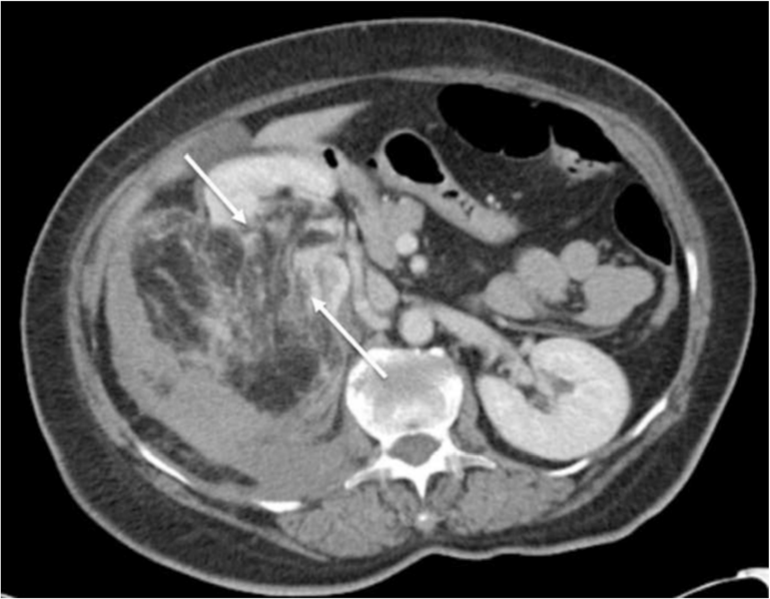
Retroperitoneal blood in the perinephric space secondary to haemorrhage from a large angiomyolipoma, evident as a fatty cleft in the right kidney (*arrows*). Only a small amount of kidney can be seen on the medial side

**Fig. 18:**
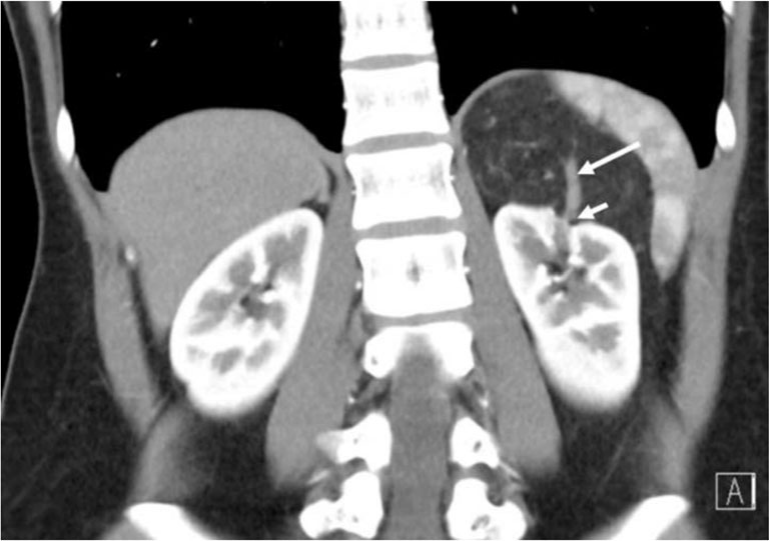
A predominantly fat-containing exophytic suprarenal angiomyolipoma. Note the small cleft (*short arrow*) in the superior pole renal cortex and the prominent vessel (*long arrow*) passing through this

Angiomyolipoma (AML) is a mesenchymal neoplasm composed of blood vessels, smooth muscle and adipose tissue [57]. AML occur sporadically in 80% of cases with a prevalence of 0.2%-0.4% or as part of an autosomal dominant condition known as tuberous sclerosis complex (TSC) and have a female predilection [63, 64]. AML’s are observed in 55%-75% of patients with TSC and present earlier in life [65].  Renal AMLs with perinephric extension are the most common macroscopic fat-containing mass in the perirenal region [66]. They are often incidental when imaging the retroperitoneal space, but do have a propensity to bleed (Fig. 17) when lesions grow larger than 4 cm or intratumoral aneurysmal change exceeds 5 mm [7, 67]. Imaging modalities are aimed at demonstrating fat within the suspected lesion, although in settings of AML and associated tuberous sclerosis, up to a third of all AMLs will be fat-poor [68]. As AML originates primarily from the renal parenchyma, it can be easily distinguished from a liposarcoma by a “beak sign” or divot at the interface of the kidney and mass, often with a perforating feeding vessel [69, 70] (Fig. 18). Calcifications are further more suggestive of liposarcoma over AML [71].

### Neoplastic

#### Solitary fibrous tumour

Solitary fibrous tumour (SFT) of the kidney is a rare entity. Initially believed to be solely a pleural malignancy, referred to as “benign fibrous mesothelioma”, SFTs can be seen anywhere throughout the body [72, 73]. Most SFTs are benign and appear as slow-growing masses in middle-aged adults, with equal frequency in men and women [74, 75]. Surgical excision is the treatment of choice, with 5-year survival close to 90% with complete excision [76]; however, complete clearance is required, as up to 20% of SFTs can exhibit malignant potential [76, 77]. SFTs on imaging display hypervascular signalling on contrast-enhanced CT. T1-weighted MR shows intermediate signalling, with flow voids on T2-weighted imaging due to dense collagen and fibrosis [53]. Intense enhancement is seen after administration of gadolinium contrast material [78].

#### Desmoid tumour

Desmoid tumours ​or aggressive fibromatosis develop from musculoaponeurotic tissue and can be sporadic or familial, such as familial adenomatous polyposis (FAP) or Gardner syndrome [46]. Desmoid tumours are hormonally responsive to oestrogen, and as such are more common in women from puberty to 40 years of age [56, 57]. Tumours usually are less than 10cm in diameter and are defined as intermediate/locally aggressive. Malignant transformation is rare [79, 80]. Histologically, desmoid tumours vary based on tissue composition (spindle cells, collagen, myxoid matrix). Imaging characteristics differ depending on the composition within the tumour, and vascularity may change with time, leading to evolving CT and MR dynamic imaging features [81]. Contrast enhancement is seen for most desmoid tumours, and heterogeneous signalling is common [82, 83]. An infiltrative border in the form of a fascial tail may be seen in up to 80% of cases on MRI [81]. T2-weighted MRI may demonstrate hypointense bands within the tumour that correlate with dense collagenous bands seen on histology [82].

#### Phaeochromocytoma

**Fig. 19:**
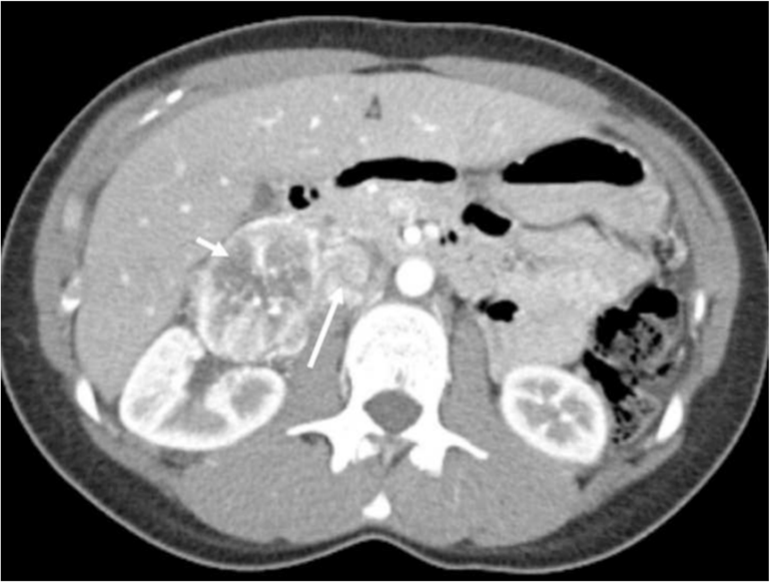
Axial portal venous CT shows a heterogeneous mass (*short arrow*) at the right renal hilum, with invasion of the inferior vena cava (*long arrow*). This was excised and was pathologically shown to be a phaeochromocytoma

Phaeochromocytomas (PCCs) and abdominal paragangliomas are rare catecholamine-producing tumours. Incidence patterns show that men and women are affected equally, in the fourth to fifth decades of life [84]. Most (90%) PCCs are located in the adrenal gland [85]. They can occur both as sporadic tumours and as a manifestation of genetic disease [86]. MRI remains the morphological imaging modality of choice in localising PCC and extra-adrenal paragangliomas, offering better contrast resolution than CT and without ionising radiation [87]. PCCs appear hypointense or iso-intense on T1-weighted imaging and markedly hyperintense on T2-weighted images. CT imaging will show a soft tissue density that enhances markedly with contrast administration, often showing areas of calcification or central necrosis [88, 89] (Fig. 19). When morphological imaging and biochemical investigations of lesions are indeterminate, functional imaging utilising meta-iodobenzylguanide (^123^I-MIBG) can achieve specificity of up to 99% and sensitivity of 77–90% in the diagnosis of PCC, and remains the most important nuclear imagining tool currently in service [89]. It is important to consider paraganglioma in appropriate cases, especially if biopsy is planned, as biopsy may lead to a catecholamine crisis characterised by headache, hypertension, sweating, haemodynamic compromise and even death. Positron emission tomography has emerged as an important diagnostic tool in adrenal malignancies due to its functional and concurrent anatomical localisation in combined PET/high-resolution CT. [^18^F]fluorodeoxy-D-glucose (FDG) when taken in by metabolically active adrenal malignancies, can localise and correlate with structural anatomy yet currently lacks the specificity required as an initial investigation of choice [87]. ^18^F-FDOPA (6-[18F]-L-fluoro-L-3,4-dihydroxyphenylalanine)-based PET/CT has shown sensitivity profiles close to 95% in diagnosing PCCs and abdominal paragangliomas, reaching 100% concordance with MRI for the detection of PCCs [90].

#### Neurofibroma

**Fig. 20:**
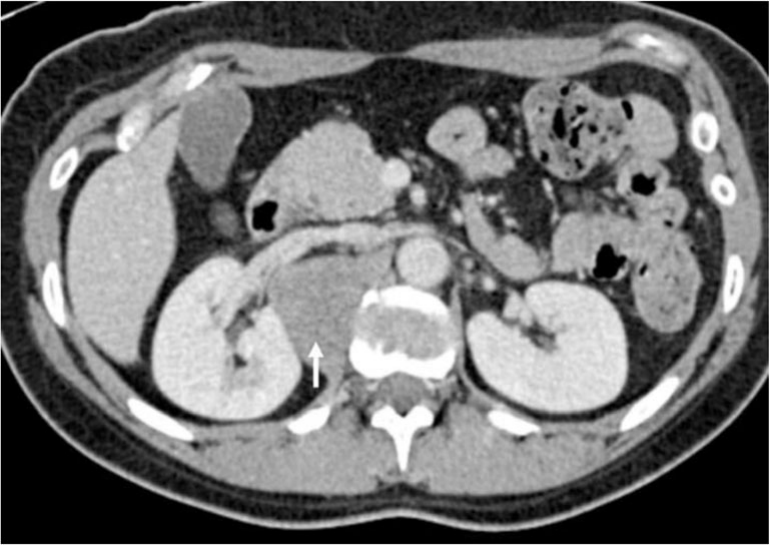
A homogenous low-density mass extending into the right perinephric space (*arrow*), with broad contact with the psoas fascia. This is a biopsy-proven neurofibroma

Neurofibroma is a benign nerve sheath tumour more often seen as an isolated entity (90%) rather than in conjunction with associated genetic conditions such as neurofibromatosis. They consist of Schwann cells, fibroblasts and rich networks of collagen fibres. More common in men, its incidence occurs in young to middle-aged men, 20 to 40 years of age [91]. On CT imaging, neurofibroma are homogenous (Fig. 20), smooth and round with distinct outlines. Imaging characteristics are secondary to their lipid rich architecture and collagen deposits [91﻿, 92]. MRI shows hypointense T1-weighted imaging consistent with neural tissue. In addition, high peripheral signalling on T2-weighted images with low central signalling can be seen due to myxoid degeneration peripherally and nerve tissue centrally [93]. When multiple neurofibroma are present, neurofibromatosis should be considered. Unlike schwannoma, neurofibroma are inseparable from the normal nerve, and surgical excision must include the adjacent nerve [94].

#### Renal cell carcinoma

**Fig. 21:**
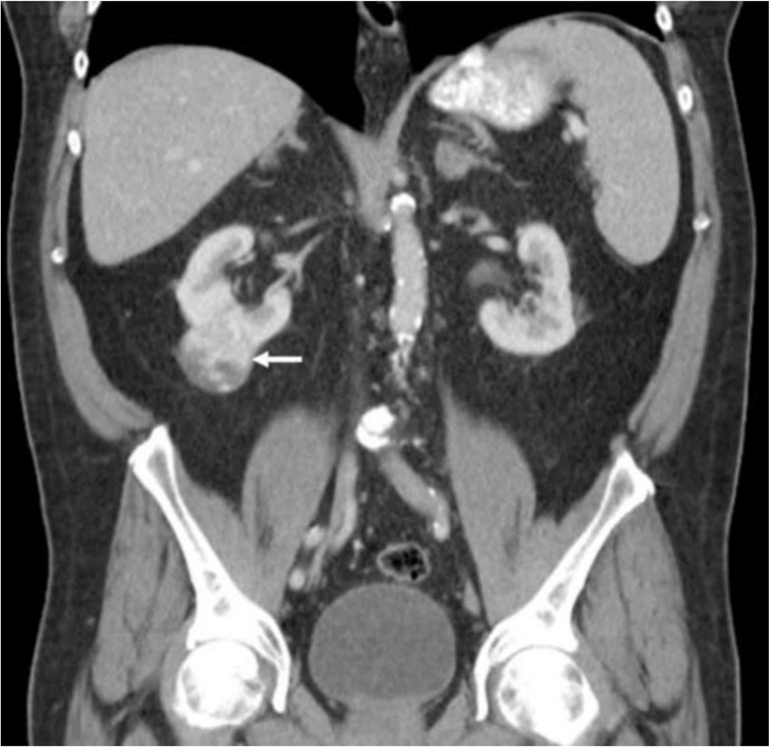
Coronal portal venous CT demonstrating an exophytic heterogeneous renal cell carcinoma arising from the lower pole of the right kidney (*arrow*)

Renal cell carcinoma (RCC) remains the most common presentation of malignancy arising from the kidney, accounting for 2–3% of all adult malignancies, with a median age at diagnosis of 65 years and a significant male predilection [95, 96]. The diagnosis of renal cell cancer encompasses a multitude of distinct cytogenetic and immunohistochemical properties that carry with them differing prognoses and imaging characteristics [97]. Clear cell subtypes remain the most frequent in presentation (75%), followed by papillary (10%) and the rarer chromophobe (5%), collecting duct and medullary subtypes [98]. Recent literature has suggested racial differences in the distribution of histological subtype [99]. The gross morphological profile of RCC can further delineate subtype, with clear cell RCC typically exhibiting exophytic growth patterns. This pattern is important as a differential for visualised masses in the perinephric space (Fig. 21). RCC that extends or spreads into the perinephric space is considered a T3 tumour and carries significant prognostic implications [100]. Other features that may differentiate histological subtype on imaging include intralesional heterogeneity, fat and enhancement patterns with contrast agents [101, 102]. The different imaging characteristics of the common histological subtypes of renal cell cancer are described in Table 2 [97, 98, 103]. Focal solid masses in the perinephric space are most commonly due to malignancy, and differential considerations include tumour extension from renal, adrenal or retroperitoneal sources [29].

**Table 2:** Renal cell histological subtypes

Subtype	Incidence	Origin	Patient age	Signal density	Biological behaviour	Post-contrast	Associations
Clear cell	75%	Proximal nephron, tubular epithelium	>50 years	Heterogeneous density/signal	Aggressive	Hypervascular	Von Hippel-Lindau (25–45%), tuberous sclerosis (2%)
Papillary	10%	Distal nephron, tubular epithelium	*>*50 years	Low T2 signal, hypodense	Aggressive	Hypovascular	Hereditary papillary RCC
Chromophobe	5%	Distal nephron	>50 years	Hypodense, intermediate signal density	Low mortality (10%)	Hypovascular	Birt-Hogg-Dubé syndrome
Collecting duct carcinoma	∼1%	Medullary collecting duct	>50 years	Heterogeneous density, low T2 signal	Highly aggressive	Hypovascular	
Medullary	∼1%	Distal nephron	20–40 years	Heterogeneous, infiltrative, low T2 signal	Extremely aggressive	Hypovascular	Sickle cell disease

#### Extra-gastrointestinal stromal tumour

Primary extra-gastrointestinal stromal tumour (EGIST) in perirenal locations is extremely rare. Gastrointestinal stromal tumour (GIST) is a non-epithelial neoplasm arising from the muscularis propria layer (interstitial cells of Cajal) of the GI tract, with characteristic exophytic growth patterns [104, 105]. Among the presentations of extra-gastrointestinal stromal tumours, most are found in the omentum and mesentery [104]. Perirenal EGISTs appear as hypovascular soft tissue masses, often non-specific, and diagnosis relies heavily on biopsy and histological analyses rather than imaging interpretation. MRI shows varying degrees of heterogeneity and signalling on both T1- and T2-weighted images, with T1-weighted imaging showing low signal intensity (iso-intense to skeletal muscle) and multiple markedly hypointense linear bands [106].

#### Metastases

**Fig. 22:**
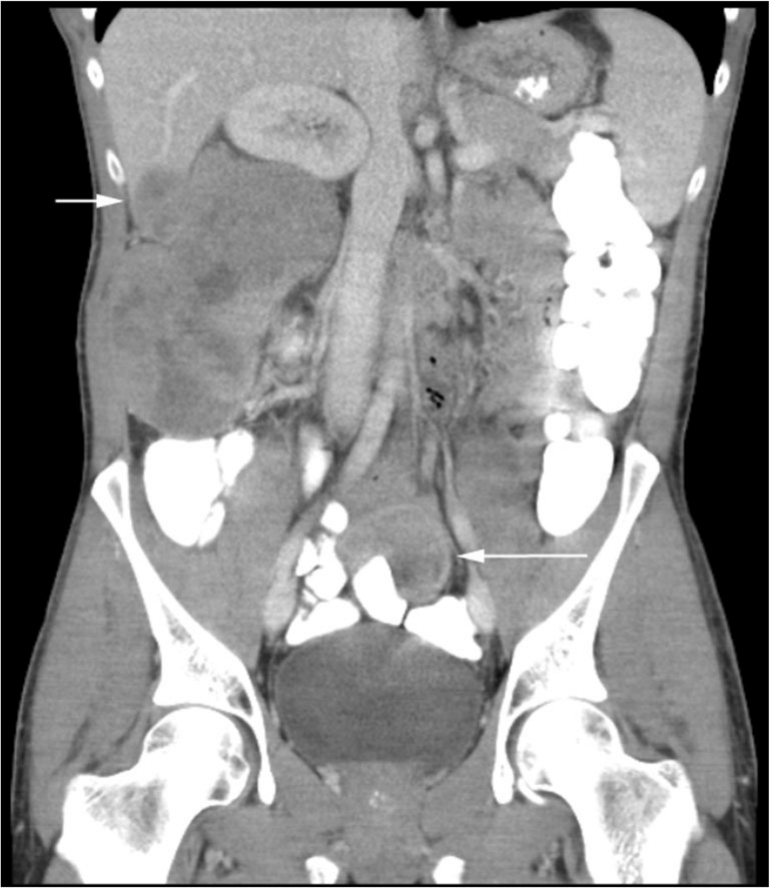
A large perinephric heterogeneous melanoma metastatic mass displaces the right kidney superiorly. A separate liver metastasis is present (*short arrow*), as is a metastasis to the small bowel (*long arrow*)

**Fig. 23:**
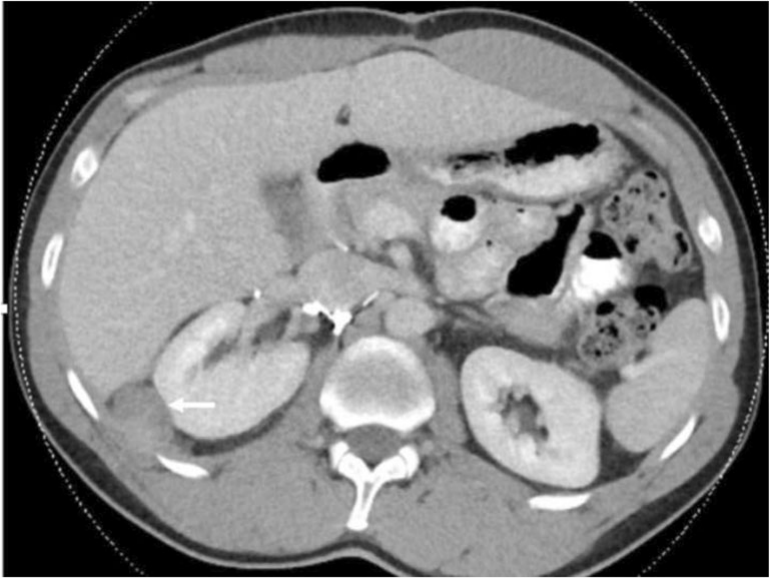
Axial portal venous phase CT shows a homogenous soft tissue metastasis in the right perinephric space, slightly indenting the adjacent kidney (*arrow*). The primary tumour, an adrenal carcinoma, had been excised previously (see clips dorsal to the inferior vena cava)

The perinephric space is an unusual site for secondary spread of malignancy. Imaging findings vary depending on the type of primary tumour. Most perinephric metastases present as multiple discrete soft tissue masses (Fig. 22). Pulmonary malignancies show a predilection for the perinephric space secondary to connections between the perirenal and mediastinal lymphatic vessels [107]. Primary metastases from melanoma, prostate, breast and gastrointestinal tumours spread via haematogenous routes, whilst pulmonary malignancies may track into the perinephric space via lymphatic spread. Metastatic spread from solid organs directly into the perinephric space can occur (Fig. 23), and local adjacent structures should be assessed for primary malignancy [29].

### Proliferative

#### Erdheim-Chester disease

**Fig. 24:**
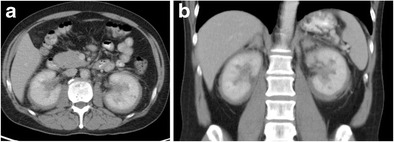
**a** A portal venous phase CT shows a soft tissue rind encasing both kidneys and extending into the renal sinus. **b** The coronal reconstruction shows underlying renal parenchyma appearing normal in this patient with Erdheim-Chester disease

Erdheim-Chester disease (lipoid granulomatosis) is a systemic non-Langerhans cell histiocytosis of unknown aetiology [108]. It affects middle-aged individuals, with no sex predilection. Perirenal involvement characteristically shows a rind-like soft tissue mass enveloping the kidney and proximal ureter (Fig. 24). MR shows low signal on both T1- and T2-weighted imaging, with minimal contrast enhancement. Progressive renal failure can occur secondary to fibrous perinephritis [108]. Treatment involves retrograde ureteral catheterisation and corticosteroid/immunosuppressive therapy until active inflammation resolves. Long bone abnormalities are crucial findings for recognition of this disease; findings, often bilateral, include cortical and medullary sclerosis and metadiaphyseal cortical thickening with sparing of the epiphyses [108, 109]. MRI can also be useful in evaluating the extent of medullary bone disease and confirming the presence of osteonecrosis [110].

#### Rosai-Dorfman disease

**Fig. 25:**
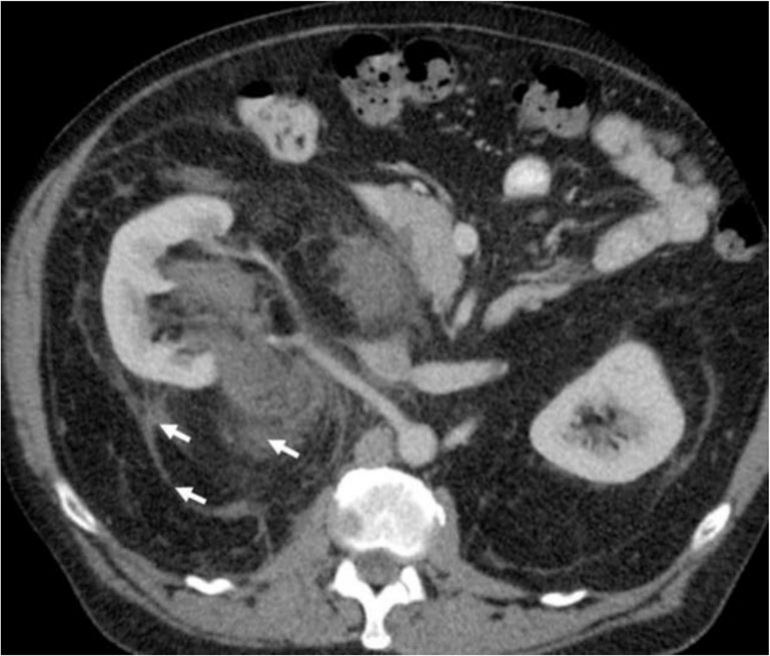
Marked expansion of the right perinephric fat with irregular soft tissue bands passing through it (*arrows*). More nodular soft tissue is present in the sinus fat and medial aspect of the perinephric space in this patient with excision-proven Rosai-Dorfman disease

Rosai-Dorfman disease (sinus histiocytosis with massive lymphadenopathy) is a proliferative benign disorder characterised by proliferation of histiocytes, with clinical features suggestive of lymphoma-like disease. The disease process tends to affect children and young adults, with extra-nodal disease present in approximately 40% [111]. Common extra-nodal sites involve the skin, head and neck, and bone. Renal involvement is rare and is seen in 4% of extra-nodal disease. CT imaging can visualise unusual hilar masses or subcapsular infiltration with varying degrees of enhancement [112, 113]. Rosai-Dorfman has been characterised by heterogeneous masses containing low-density elements almost approaching fat, with marked expansion of the perinephric space (Fig. 25); the kidneys may also be displaced and may show evidence of subcapsular infiltration [114].

## Abbreviations

CT: Computed tomography
MRI: Magnetic resonance imaging
PET: Positron emission tomography
APPFC: Acute peripancreatic fluid collection
AML: Angiomyolipoma
SFT: Solitary fibrous tumour
PCC: Phaeochromocytoma
I-MIBG: ^123^meta-iodobenzylguanide
FDG: [^18^F]fluorodeoxy-D-glucose
F-FDOPA: 6-[^18^F]L-fluoro-L-3,4-dihydroxyphenylalanine
EGIST: Extra-gastrointestinal stromal tumour
